# Supplementary material for: Habitat loss estimation for assessing terrestrial mammalian species extinction risk: an open data framework
Source: PeerJ. 2022 Dec 12;10:e14289. doi: 10.7717/peerj.14289 (PMC9753759; doi:10.7717/peerj.14289)
Supplement: Supplemental Information 1 [file peerj-10-14289-s001.docx]

**Supplemental File 1:** Description of output table columns.

| **Output parameter** | **Definition** |
| --- | --- |
| **FY_sqkm** | Total habitat in the first year, according to the taxon’s time-window, in square kilometers (= sum of all pixels equal 1). |
| **First_year** | First year of assessment, according to the taxon’s time-window. |
| **Habitat_classes** | Classes defined as habitat, according to the taxon’s biology. |
| **Higher_elev** | Higher elevation – to be used if the species has elevation constraints |
| **LY_sqkm** | Total habitat in the Last year, according to the taxon’s time-window, in square kilometer (= sum of all pixels equal 1). |
| **Last_year** | Last year of assessment, according to the taxon’s time-window. |
| **Lower_elev** | Lower elevation – to be used if the species has some elevation constrains |
| **Perc_loss** | Percentage of habitat loss, in the defined time-window and distribution area. |
| **Scale** | Scale of the raster files (the default value is 30 m). |
| **Species** | Name of the target species. |


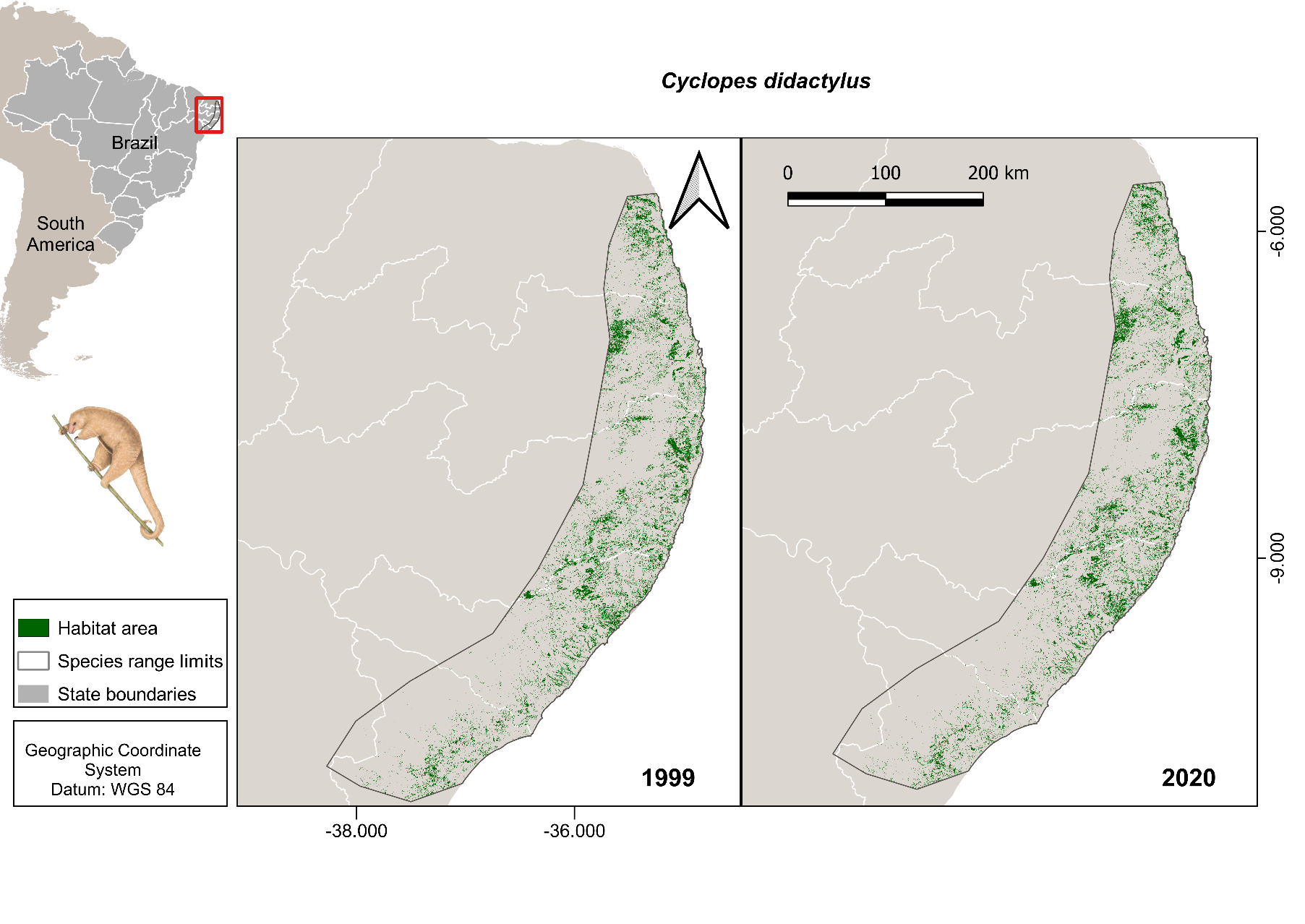


**Figure S1:** Illustration of an example of habitat increasing in the northeast distribution of *Cyclopes didactylus.*

*
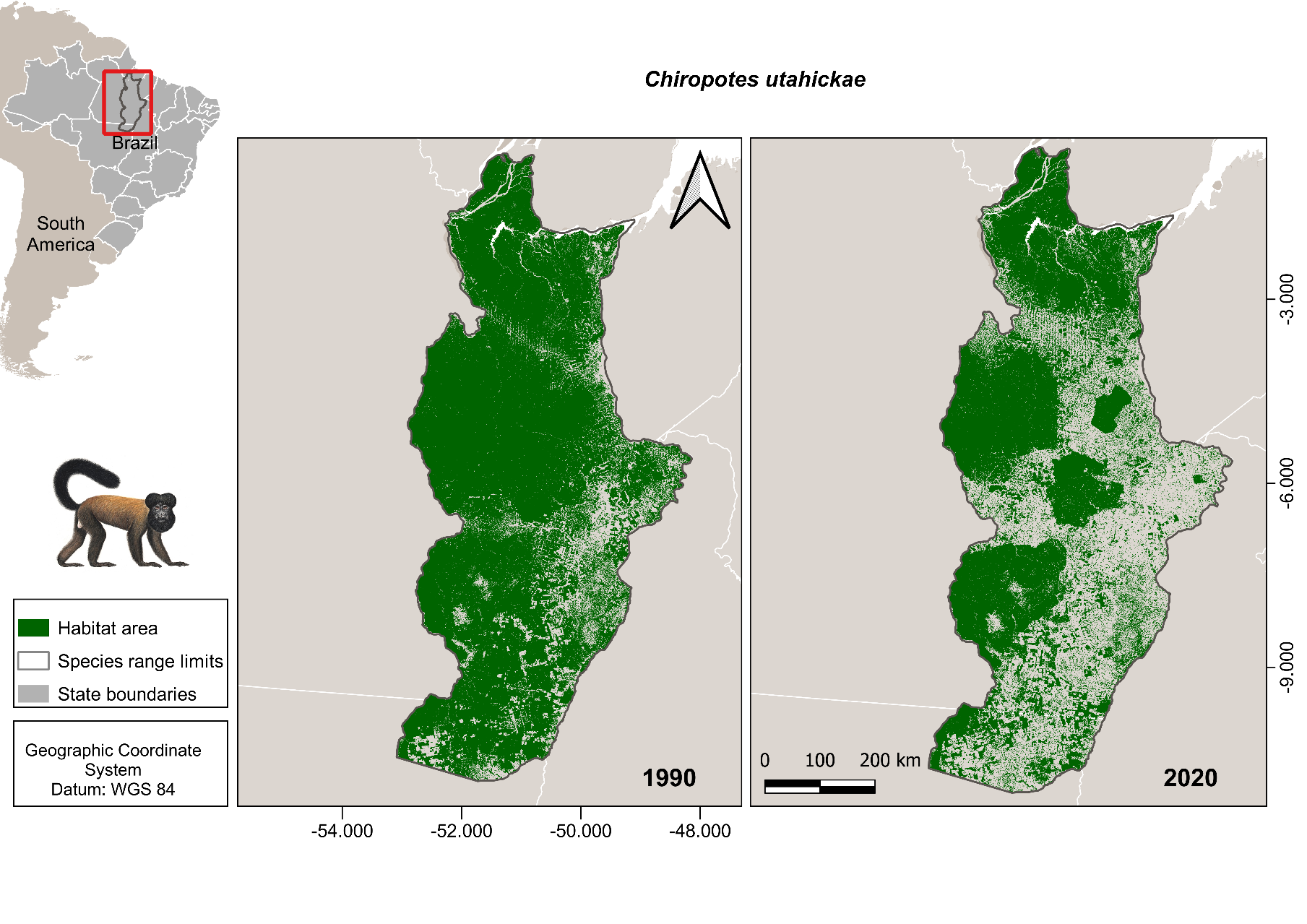
*

**Figure S2:** Pattern of habitat loss in the form of a regular geometric for *Chiropotes utahicke*.


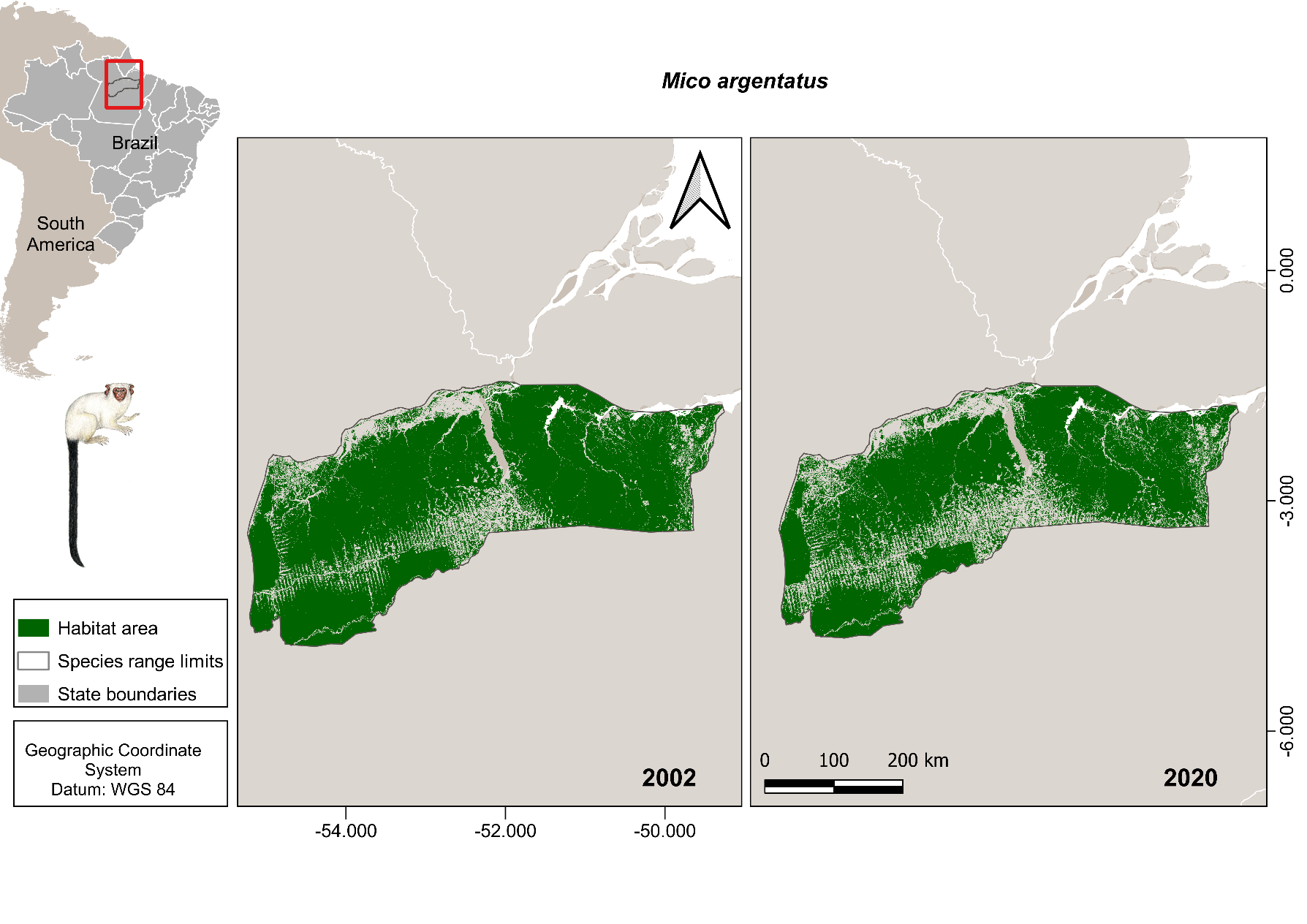


**Figure S3:** Pattern of habitat loss in the form of a herringbone for *Mico argentatus*.


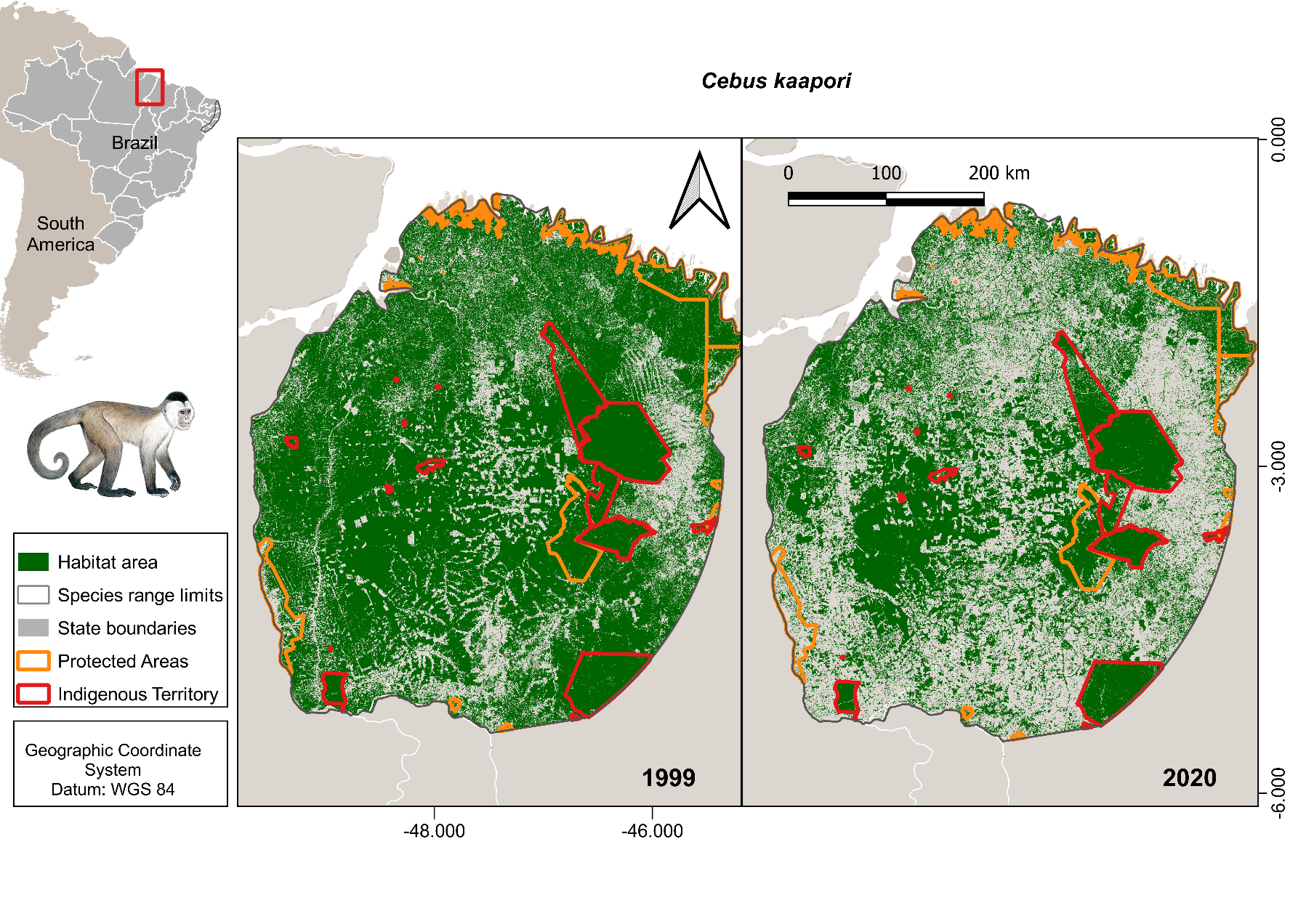


**Figure S4:** An illustration of the importance of Indigenous Territories and Protected Areas for the conservation of *Cebus kaapori* habitats.
