# Supplementary figures and images for: Habitat loss estimation for assessing terrestrial mammalian species extinction risk: an open data framework
Source: PeerJ. 2022 Dec 12;10:e14289. doi: 10.7717/peerj.14289 (PMC9753759; doi:10.7717/peerj.14289)

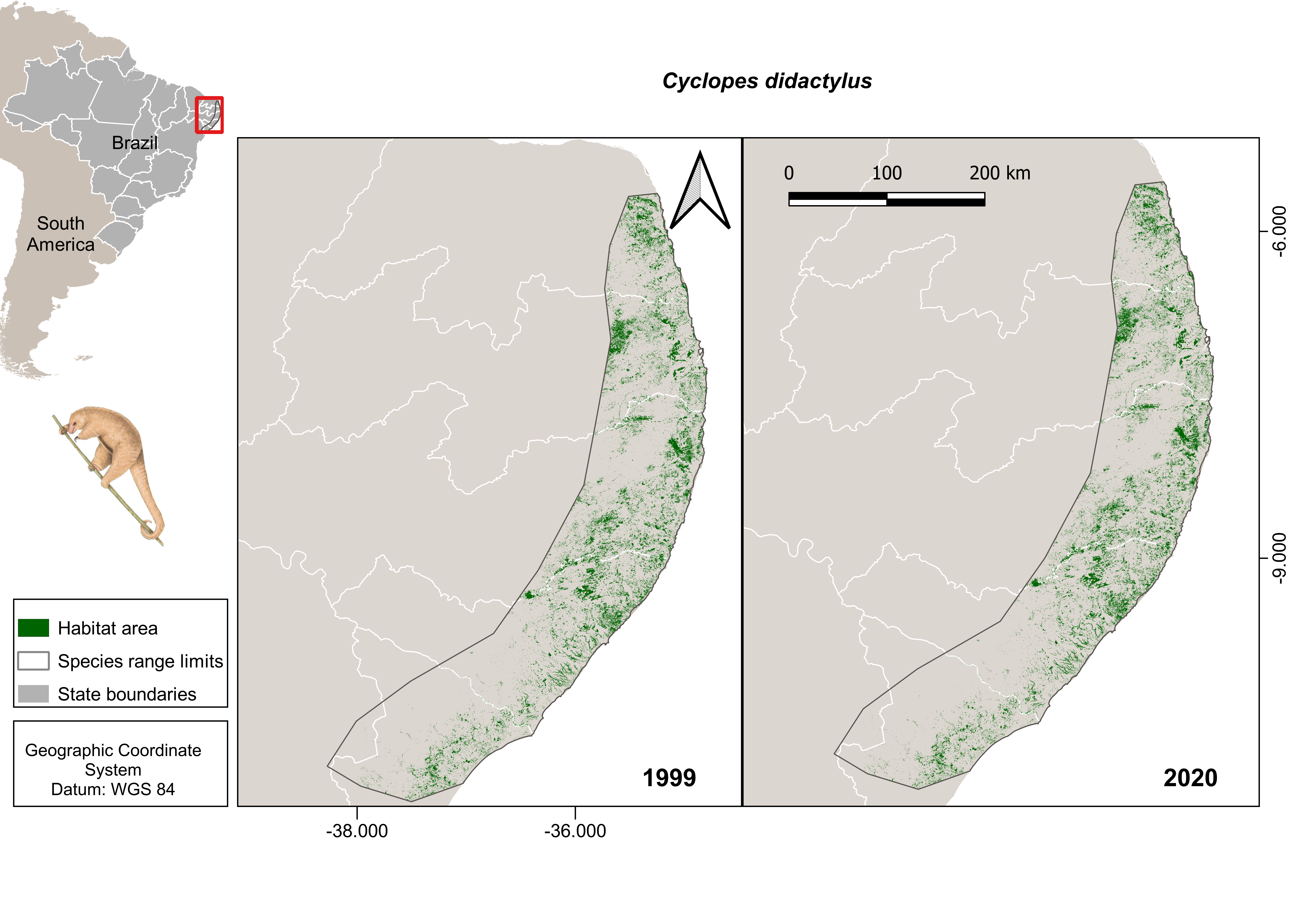

Supplement: Supplemental Information 3 [file peerj-10-14289-s003.png]

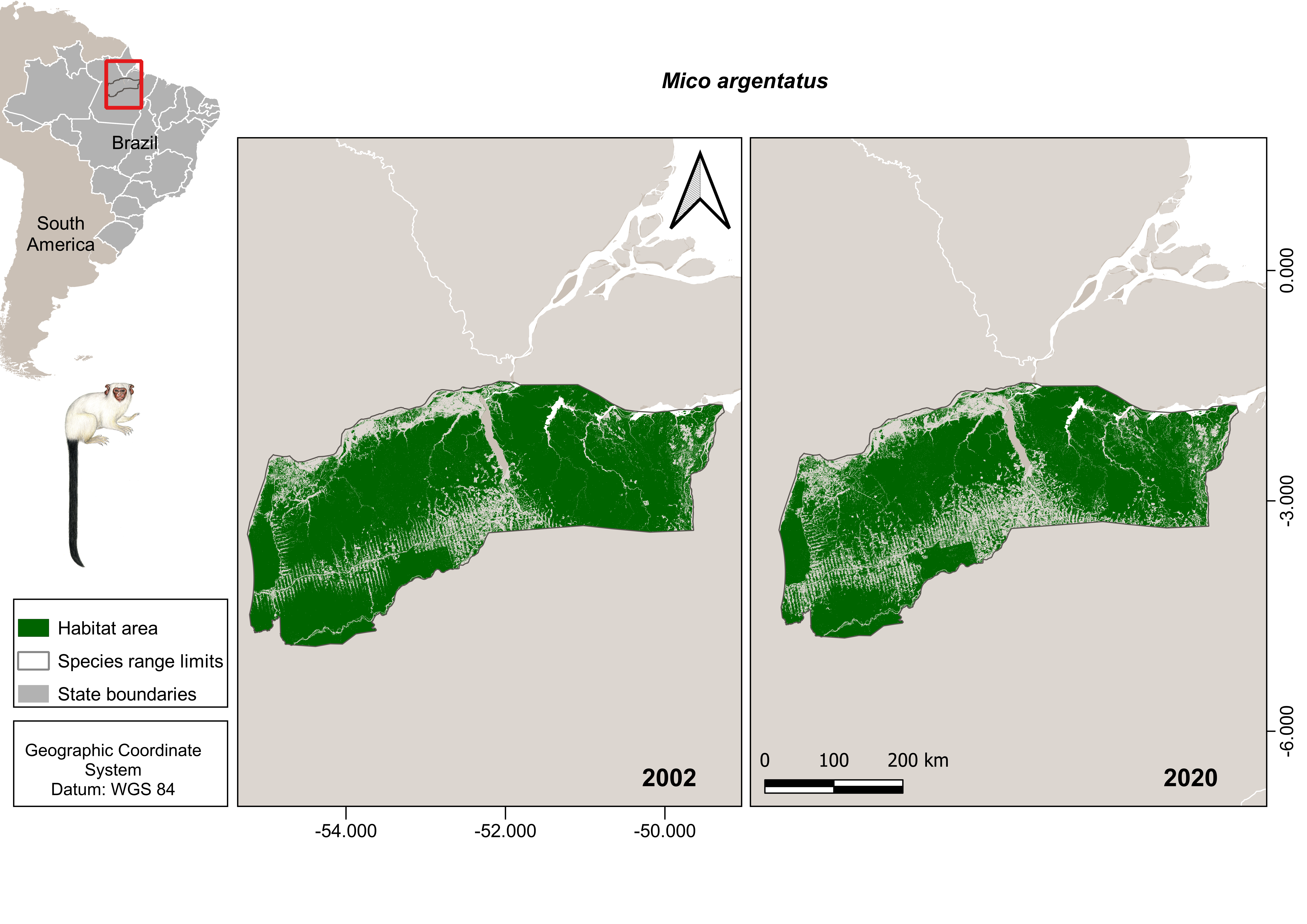

Supplement: Supplemental Information 4 [file peerj-10-14289-s004.png]

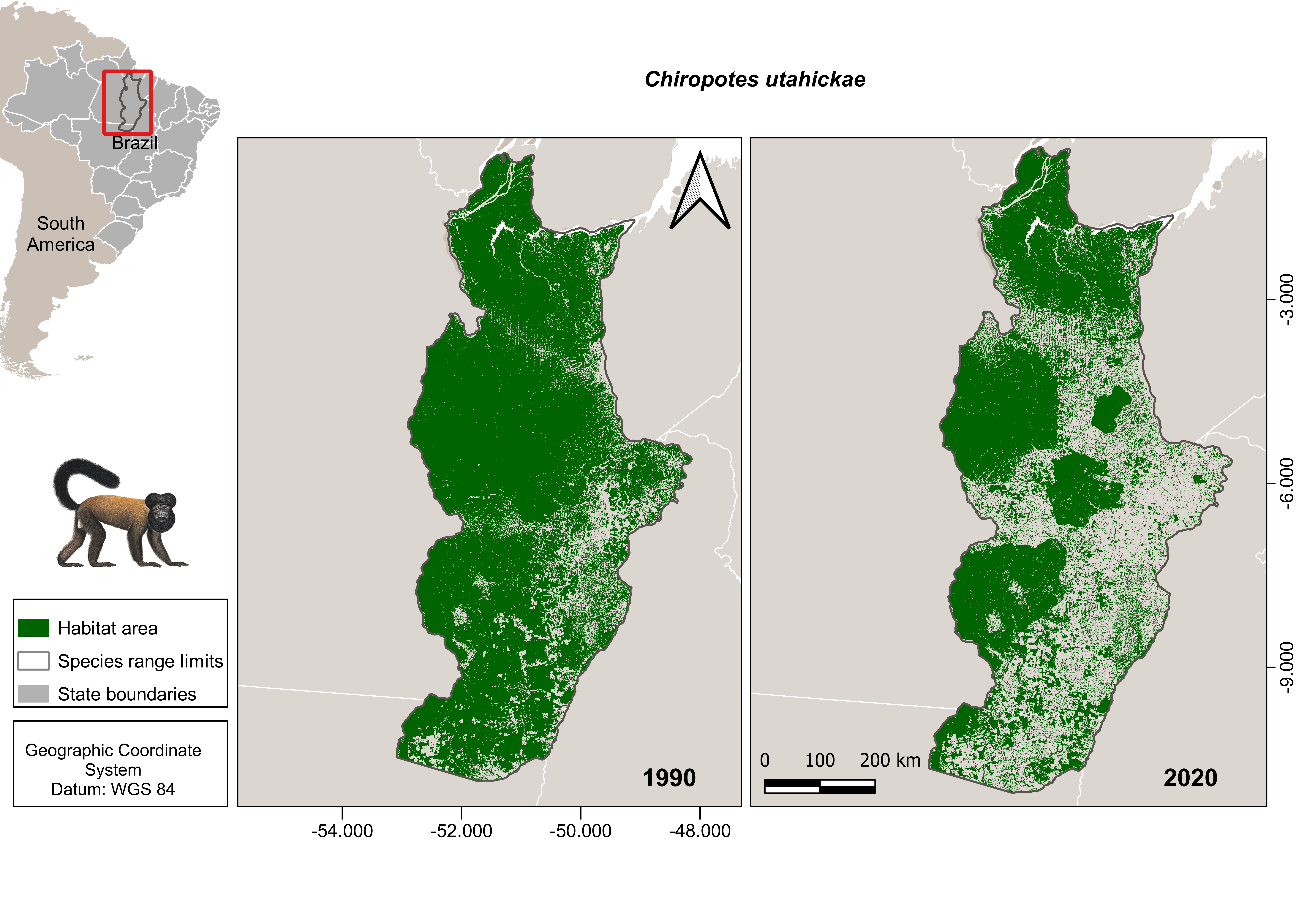

Supplement: Supplemental Information 5 [file peerj-10-14289-s005.png]

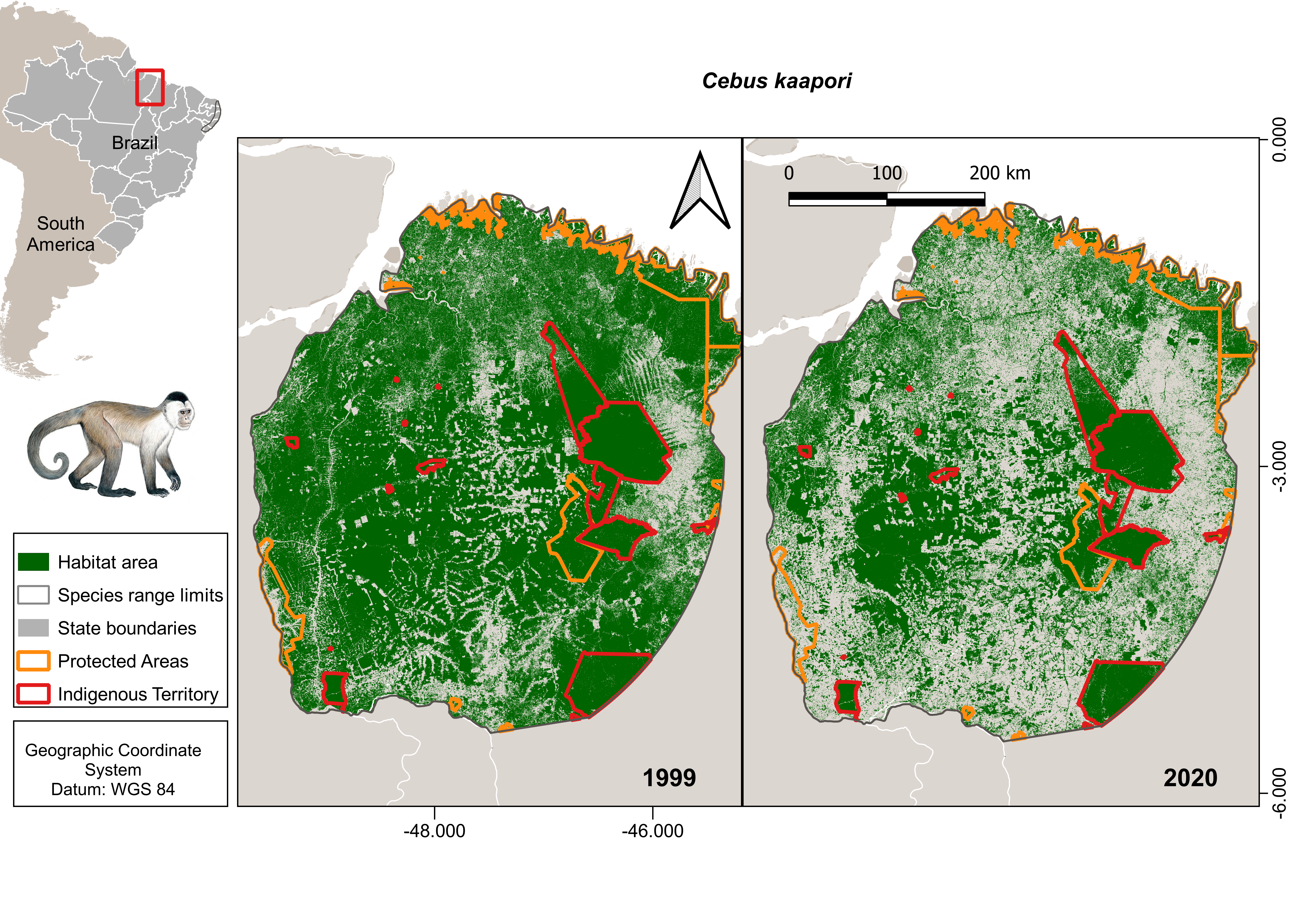

Supplement: Supplemental Information 6 [file peerj-10-14289-s006.png]
